# Supplementary material for: Distinct adaptive mechanisms drive recovery from aneuploidy caused by loss of the Ulp2 SUMO protease
Source: Nat Commun. 2018 Dec 21;9:5417. doi: 10.1038/s41467-018-07836-0 (PMC6303320; doi:10.1038/s41467-018-07836-0)
Supplement: Supplementary file 1 — Supplementary Information [file 41467_2018_7836_MOESM1_ESM.pdf]

# Distinct Adaptive Mechanisms Drive Recovery from Aneuploidy Caused by Loss of the Ulp2 SUMO Protease

Ryu et al.

## Supplementary Figures

## Supplementary Fig. 1

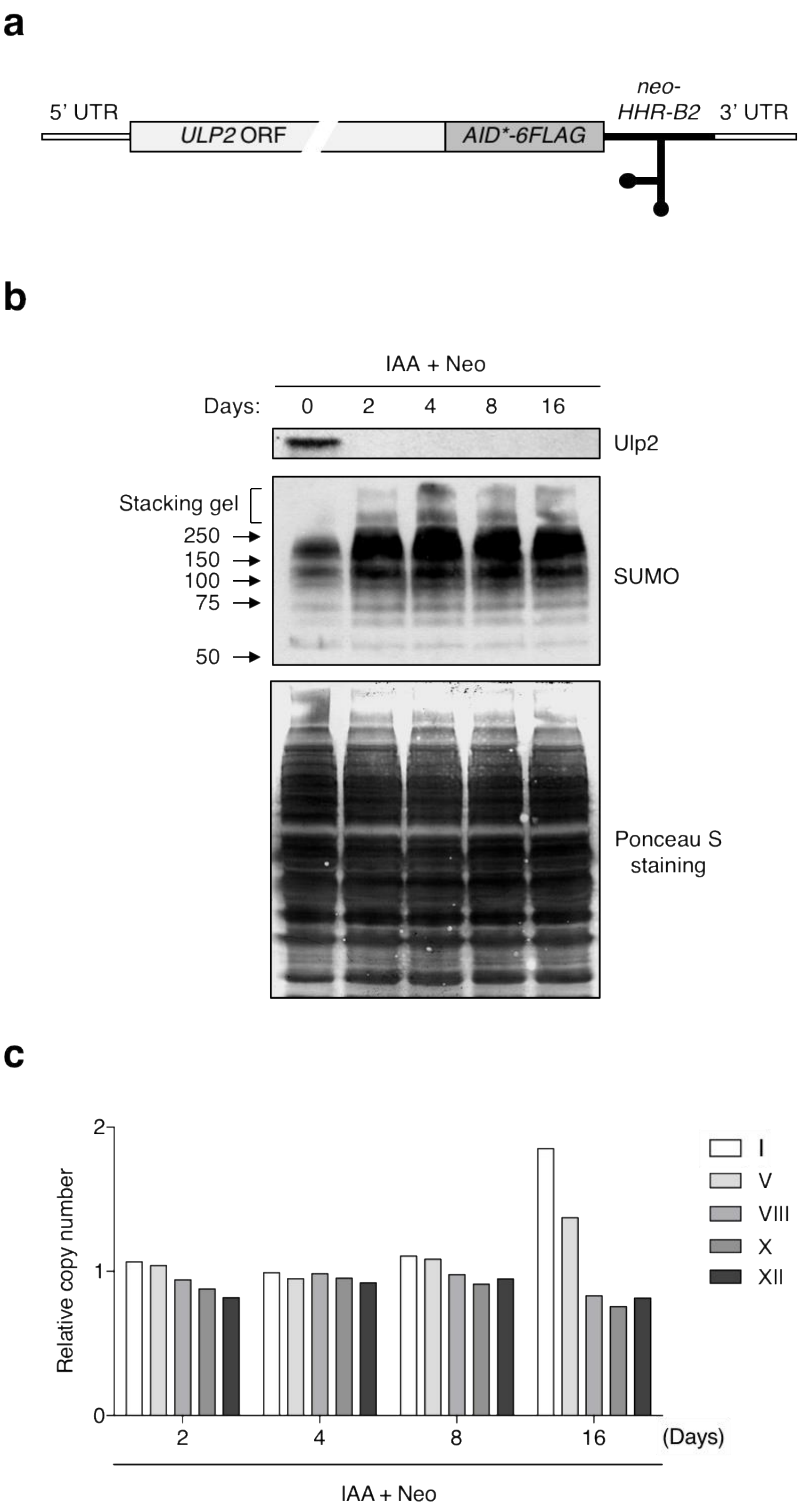

**Supplementary Fig. 1** ChrI disomy was generated after 16 days in Ulp2 protein continuously depleted cells.

**a** Schematic diagram of *ULP2-AID*-6FLAG-neo-HHR-B2*. Gray and dark gray boxes indicate *ULP2* ORF and *AID*-6FlAG* tag, respectively. Neomycin-inducible hammerhead ribozyme (HHR) in the 3′ UTR of *ULP2* is represented by black color.

**b** Immunoblotting with anti-Flag antibody to detect Ulp2-AID*-6Flag and anti-SUMO antibody to detect sumoylated proteins in extracts prepared from the strains containing *ULP2-AID*-6FLAG-neo-HHR-B2*. Cells were incubated in YPD medium with 500 μM indoleacetic acid (IAA) and 100 μg/ml of neomycin, and the cultures were diluted daily with fresh YPD containing IAA and neomycin.

**c** Aneuploidy analysis of cells from panel A as in Fig. 4b. The results were normalized to chromosome copy number from day 0 cells harvested at the onset of IAA and neomycin treatment.

**Supplementary Fig. 2**

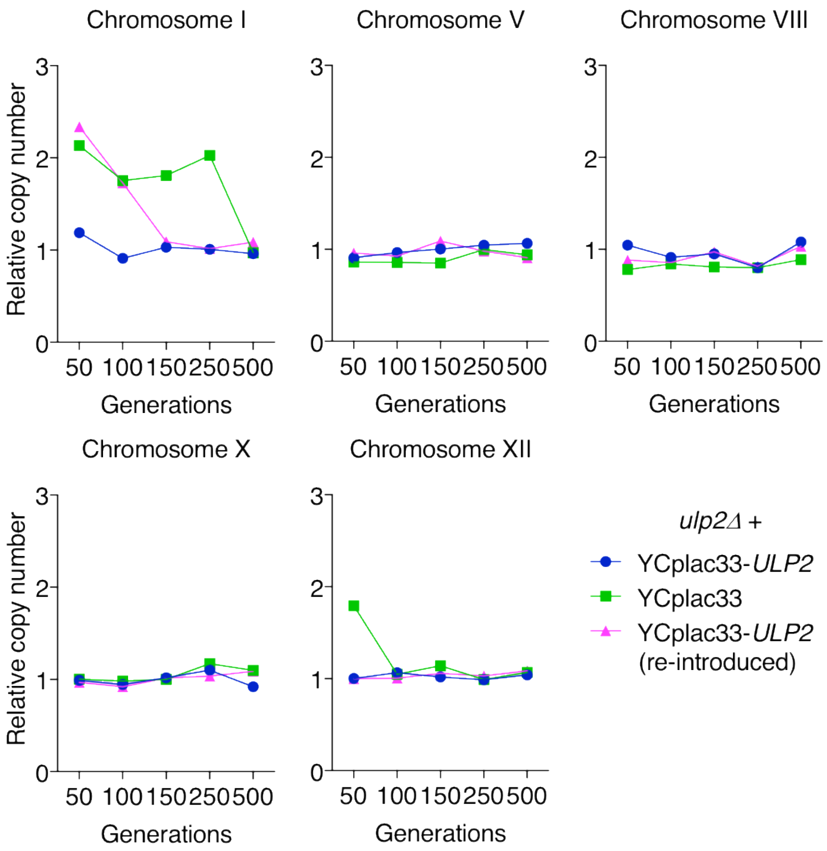


**Supplementary Fig. 2** The aneuploidy of *ulp2Δ* cells was reversed during laboratory cell evolution.

Chromosome copy number of *ulp2Δ* strains was monitored as shown in Fig. 1b.

## Supplementary Fig. 3

**a**

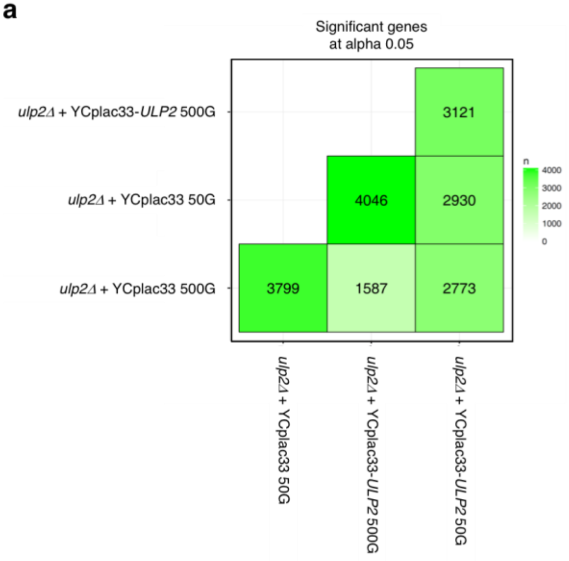


**b**

Significantly differentially expressed (SDE) genes

| Strain | Upregulated | Downregulated |
|---|---|---|
| *ulp2Δ* + YCplac33-*ULP2* 500G | 122 | 157 |
| *ulp2Δ* + YCplac33 50G | 250 | 69 |
| *ulp2Δ* + YCplac33 500G | 134 | 92 |

**Supplementary Fig. 3** Levels of many RNAs were significantly different in *ulp2Δ* strains following *in vitro* evolution.

**a** A large portion of transcripts have significantly different levels (alpha value less than 0.05) when compared between the pairs of strains.

**b** Table of significantly differentially expressed (SDE) genes that are upregulated or downregulated in the indicated mutant strain as compared to MHY1379 (*ulp2Δ* + YCplac33-*ULP2*) 50G.

**Supplementary Fig. 4**

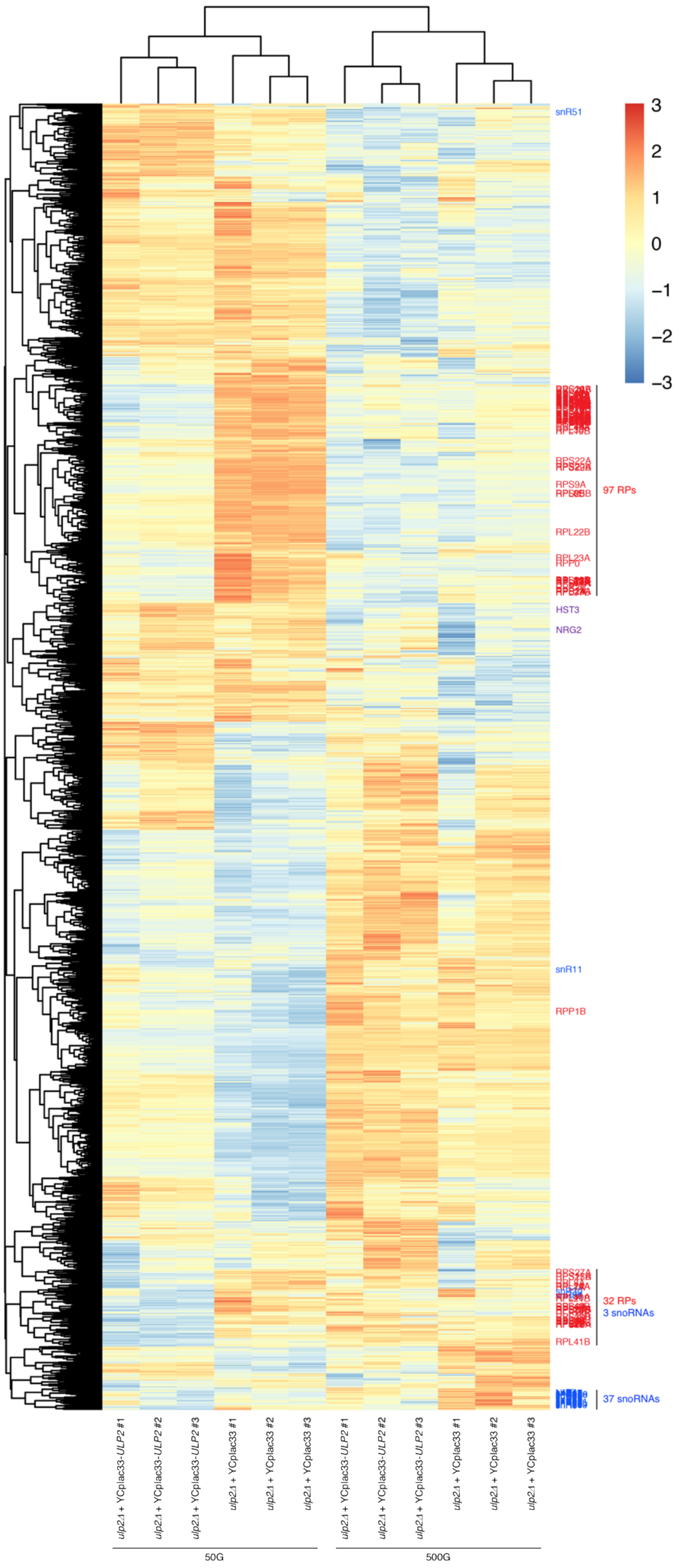

**Supplementary Fig. 4** Transcriptome analysis of *ulp2Δ* strains during laboratory evolution. Two-dimensional agglomerative hierarchical clustering of significantly up- or down-regulated (5,271) genes ($P < 0.05$) in triplicate RNA samples. Ribosomal protein (RP) genes highly expressed in *ulp2Δ* + YCplac33 50G, as compared to MHY1379 (*ulp2Δ* + YCplac33-*ULP2*) 50G, are marked in red color at right. *HST3*/*NRG2* downregulated transcripts and snoRNAs upregulated in *ulp2Δ* + YCplac33 500G are marked with purple and blue colors, respectively.

**Supplementary Fig. 5**

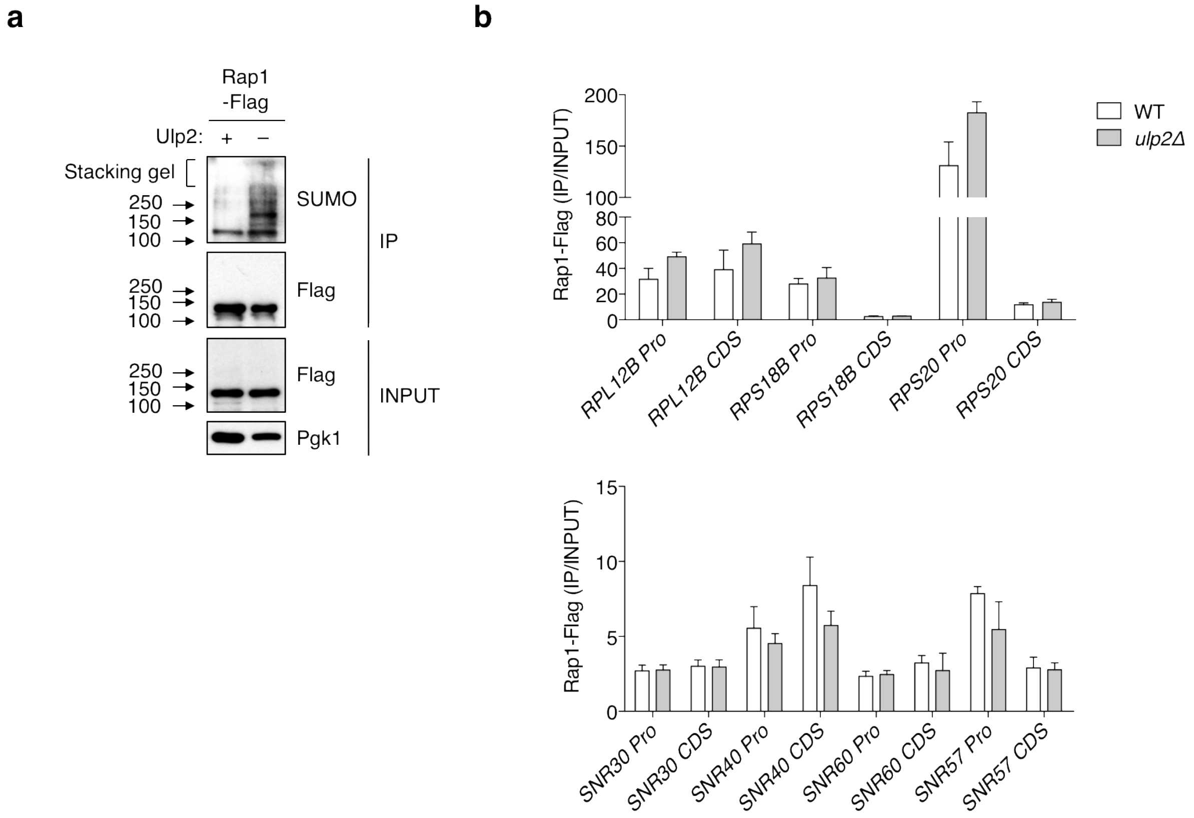


**Supplementary Fig. 5** Ulp2 reduces (poly)SUMO-Rap1 levels, but it does not appear to affect recruitment of Rap1 to RP and snoRNA genes.

**a** Immunoprecipitation (IP) of Rap1-Flag with anti-Flag agarose from denatured yeast extracts in WT and *ulp2Δ* strains expressing Flag-tagged Rap1 followed by immunoblot analysis with anti-SUMO or anti-Flag antibodies. Anti-PGK was used as a loading control for protein input.

**b** ChIP analysis of WT and *ulp2Δ* strains expressing Flag-tagged Rap1 as in Fig. 2a**.** The error bars indicate the SD from two experiments.

## Supplementary Fig. 6

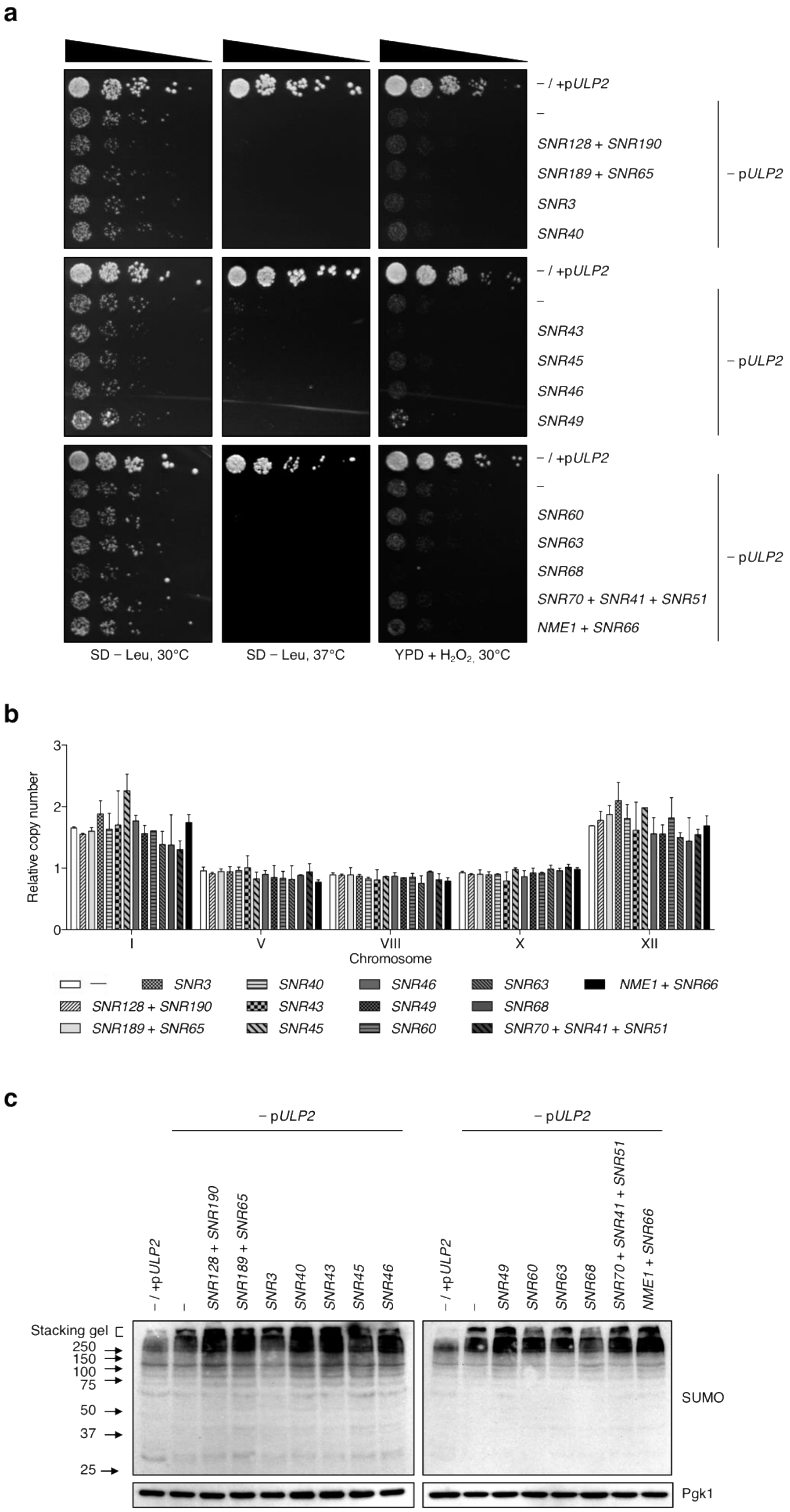

**Supplementary Fig. 6** High-copy expression of the indicated individual snoRNAs has no effect on the growth defects, aneuploidy or accumulation of high molecular weight SUMO conjugates caused by acute loss of Ulp2.

**a** Growth assays of yeast transformants. After spotting cells in five-fold serial dilutions on the indicated plates, the plates were incubated for 2-5 days at the temperatures shown.

**b** qPCR ploidy assays were performed in the indicated strains as in Fig. 4b.

**c** Immunoblot analysis of SUMO-conjugated proteins in extracts from the indicated strains as in Fig. 1e.

# Supplementary Fig. 7

**a**

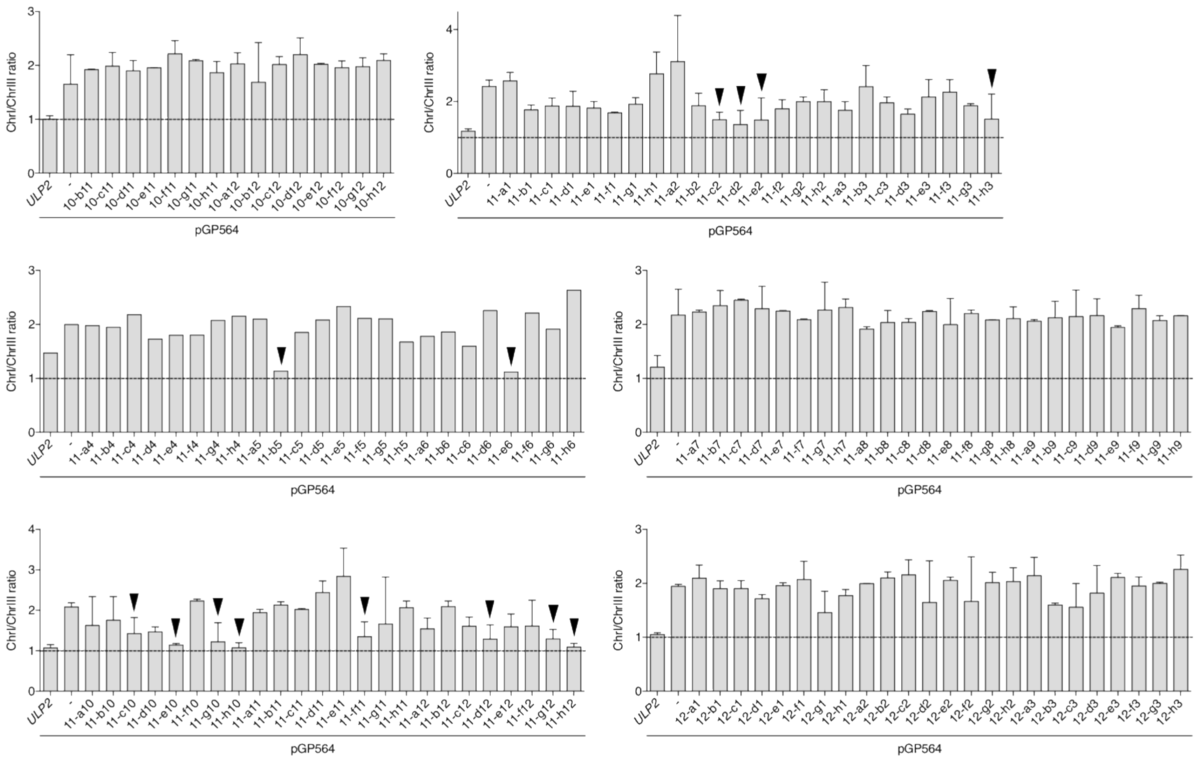


**b**

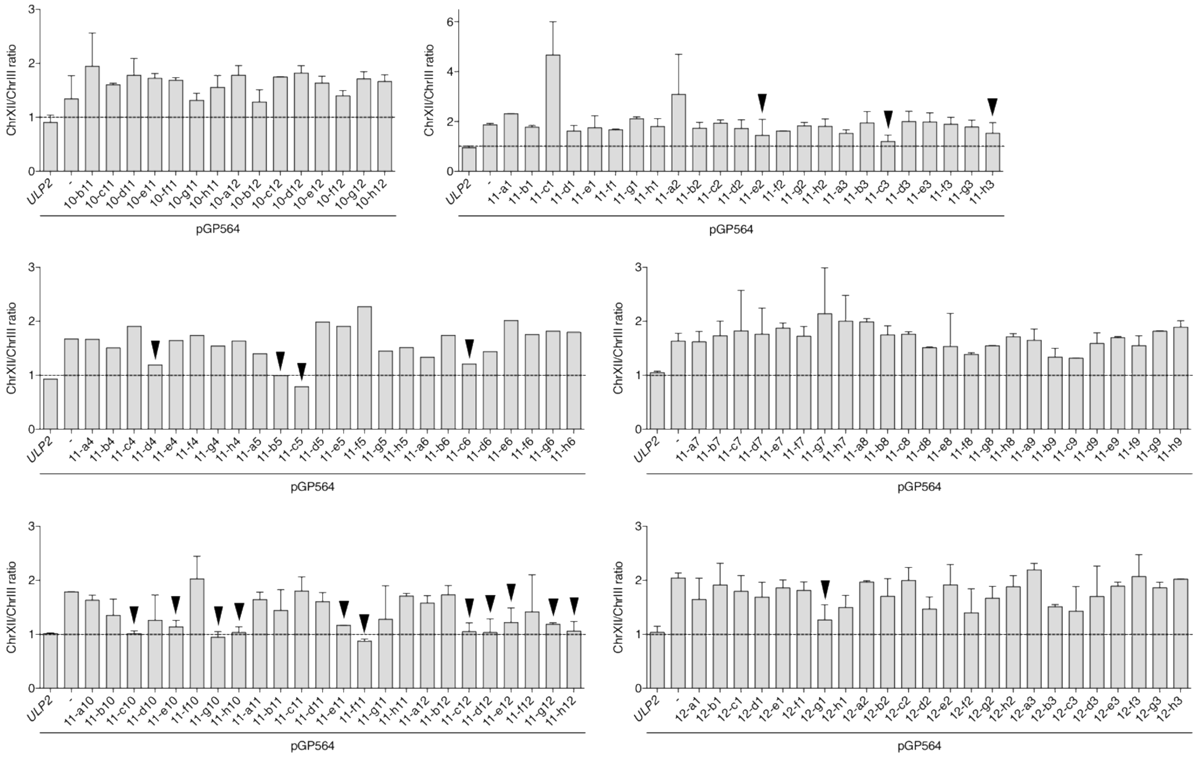

**Supplementary Fig. 7** Screening of ChrXII genes for suppression of *ulp2Δ* aneuploidy

**a**, **b** qPCR ploidy assays were performed with *ulp2Δ* strains containing the indicated 2-μm plasmids. The ratios of ChrI (**a**) and ChrXII (**b**) to ChrIII were both analyzed in duplicate qPCR reactions. The error bars indicate the SD from two experiments. Arrowheads indicate candidate plasmids showing a possible change of *ulp2Δ* aneuploidy.

**Supplementary Fig. 8**

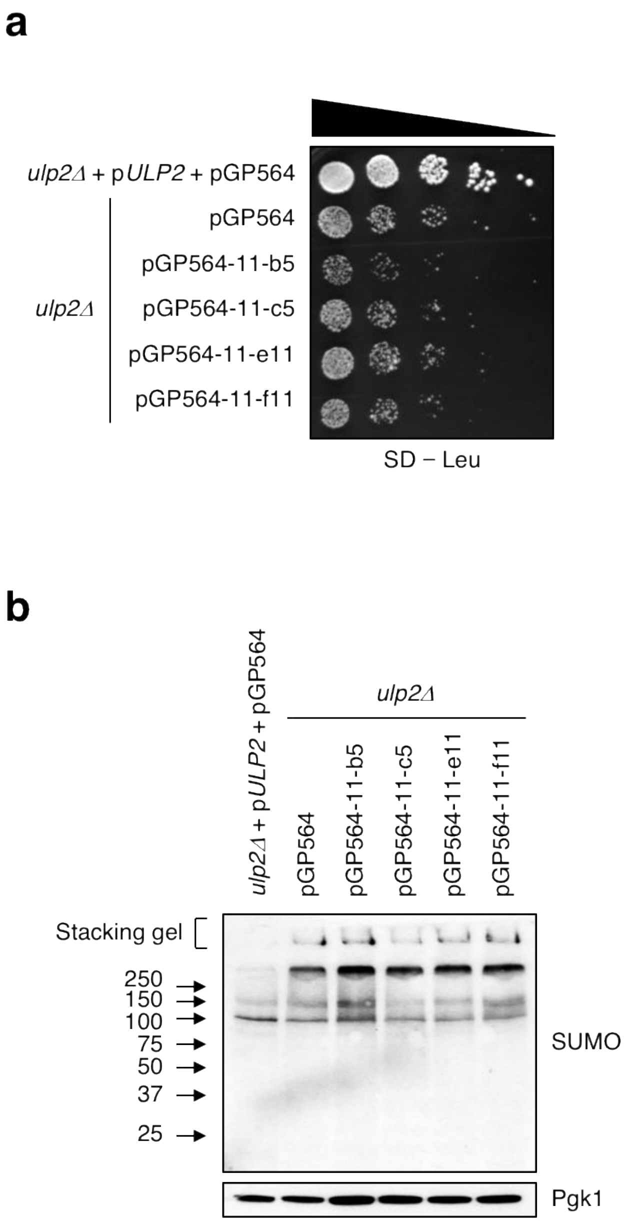


**Supplementary Fig. 8** Plasmids identified that suppressed *ulp2Δ* ChrXII disomy did not suppress the growth defect or accumulation of polySUMO conjugates in cells lacking *ULP2*.

**a** Growth assay of the indicated yeast transformants. After spotting cells in five-fold serial dilutions on an SD – Leu plate, the plate was incubated for 2 days at 30°C.

**b** Immunoblot analysis of SUMO conjugates in extracts prepared from the indicated strains as in Fig. 1e.

**Supplementary Fig. 9**

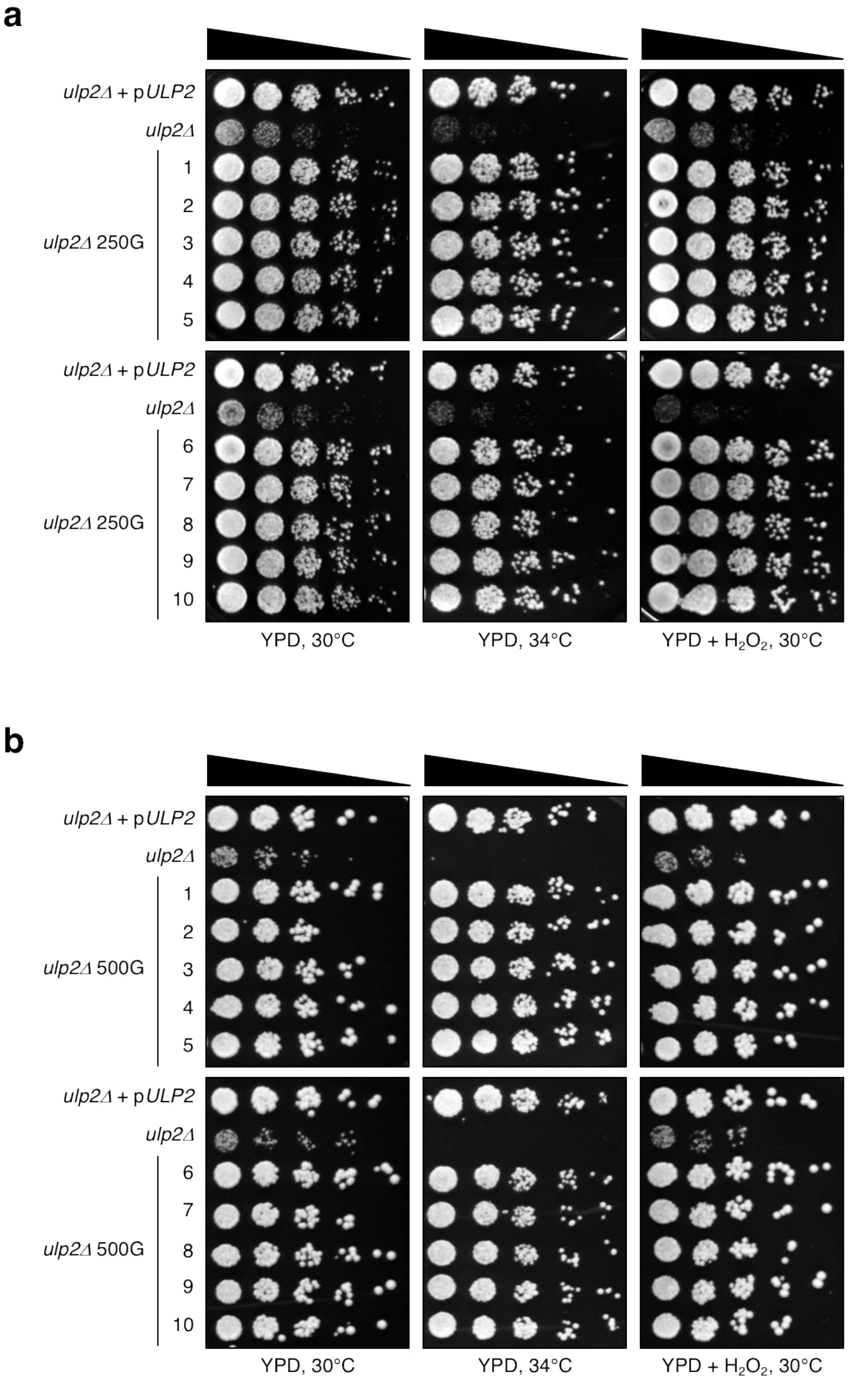


**Supplementary Fig. 9** *ulp2Δ* cells become resistant to stress following high passage.

**a**, **b** Growth assay of the indicated strains passaged for 250 (**a**) or 500 (**b**) generations. After spotting cells in five-fold serial dilutions on the indicated plates, they were incubated for 2-3 days at 30°C or 34°C, as shown.

**Supplementary Fig. 10**

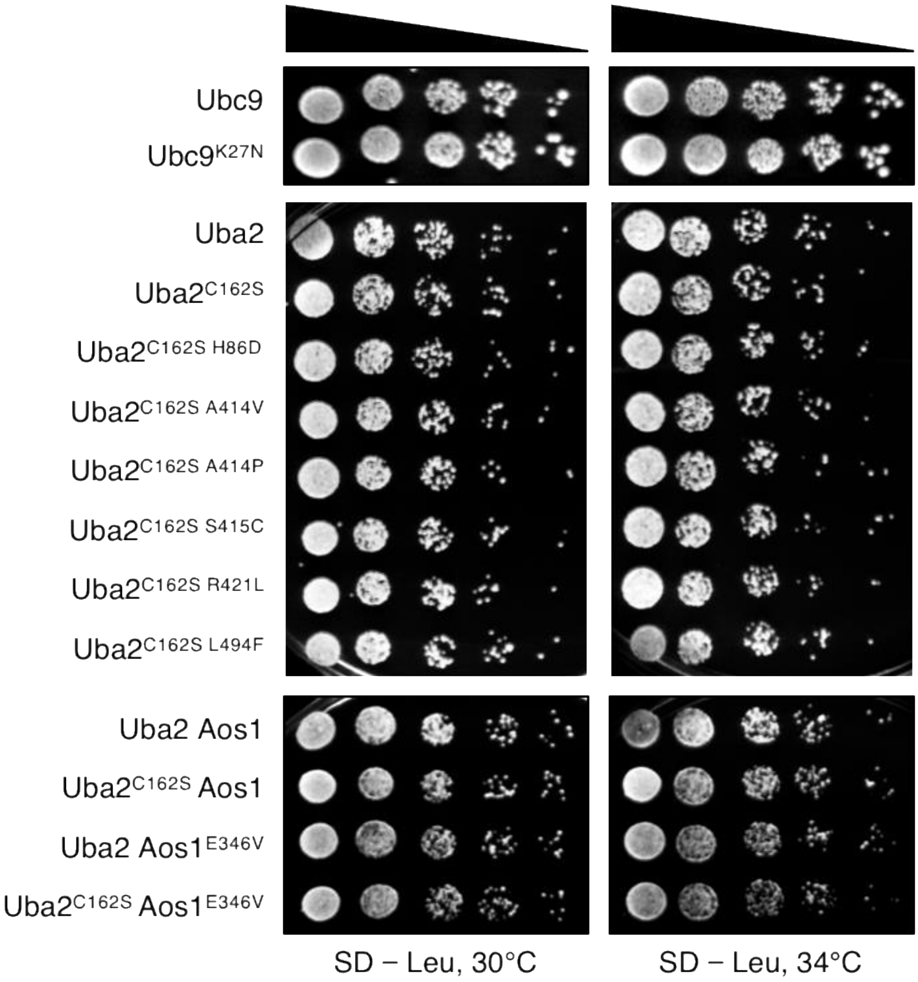


**Supplementary Fig. 10** Identified mutations of Ubc9, Uba2 and Aos1 do not affect cell growth. Growth assay of strains expressing the indicated proteins; the strains have a normal copy of *ULP2*. After spotting cells in five-fold serial dilutions, the SD – Leu plates were incubated for 2 days at 30°C or 34°C.

**Supplementary Fig. 11** Original WB Figures**.**

## Fig. 1d

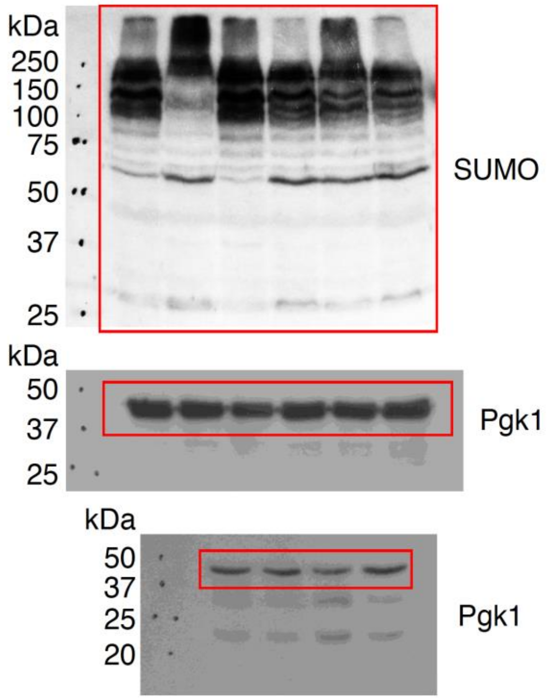


## Fig. 2d

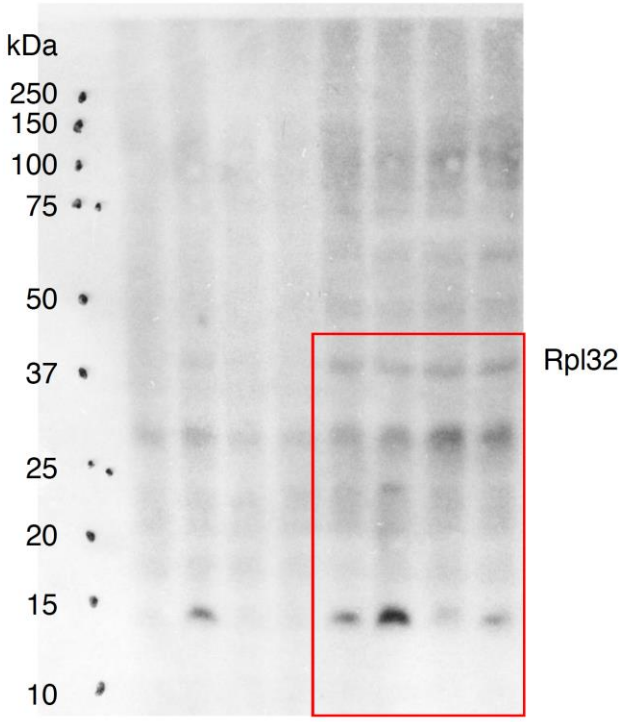


## Fig. 2h

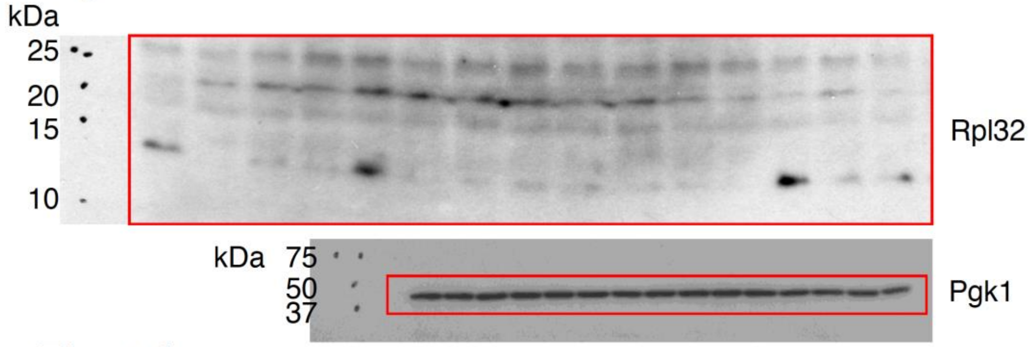


## Fig. 3f

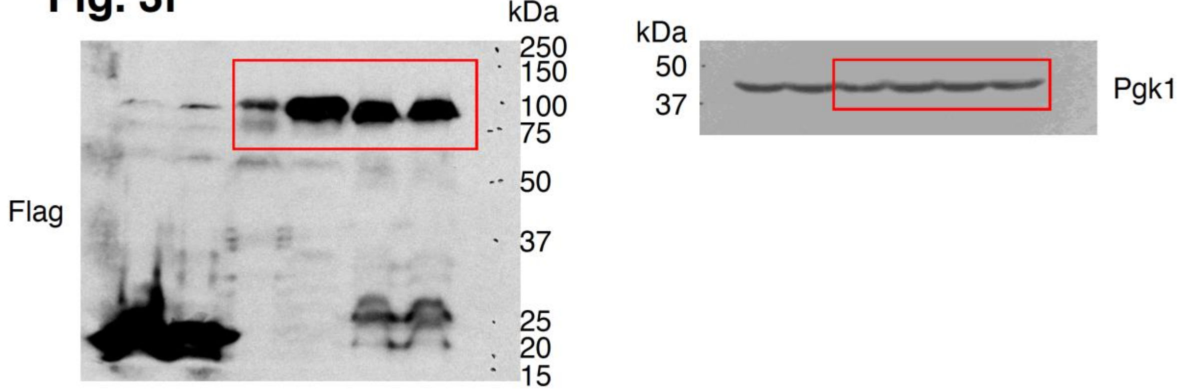

**Fig. 5e**

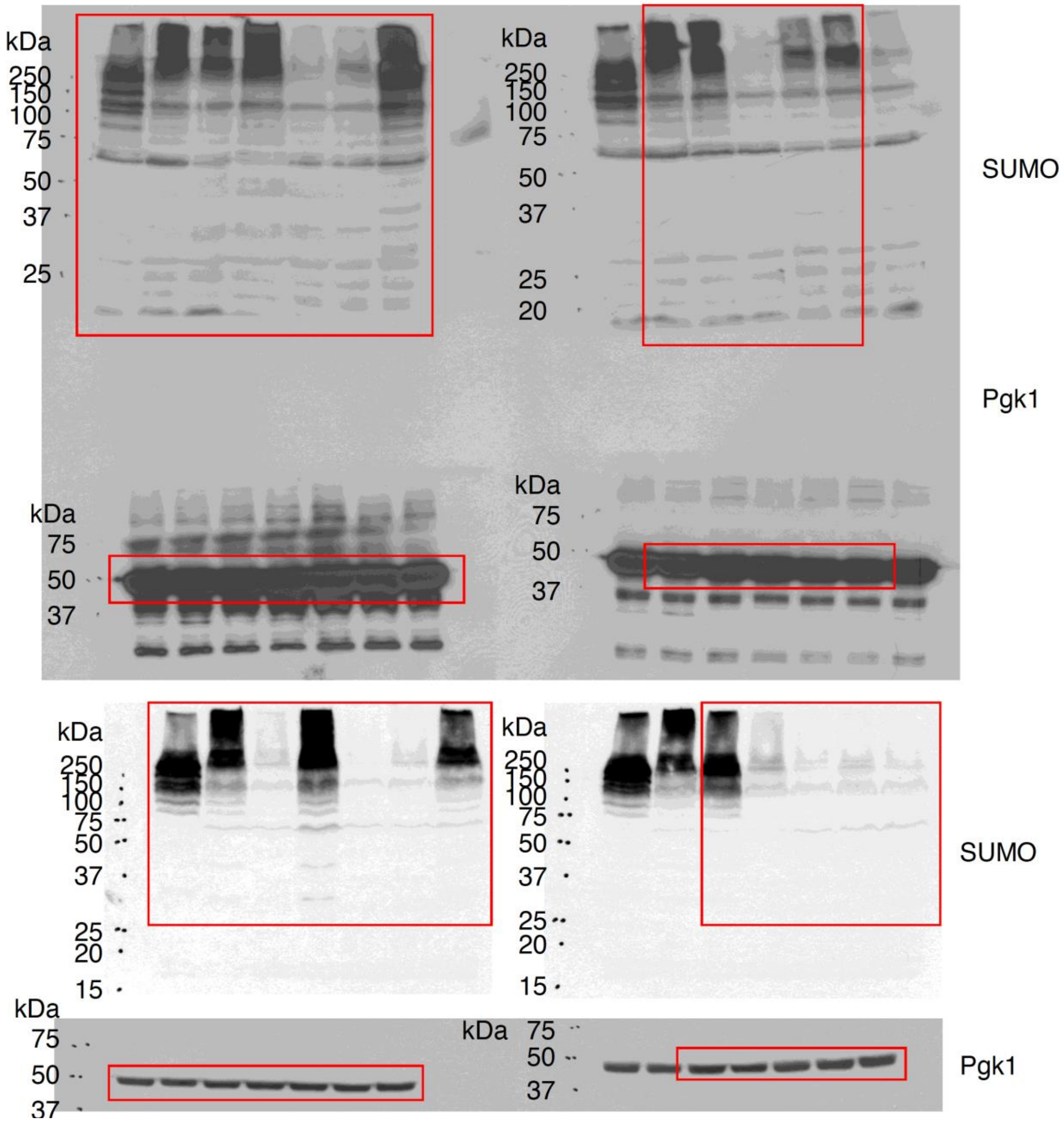

Fig. 5g

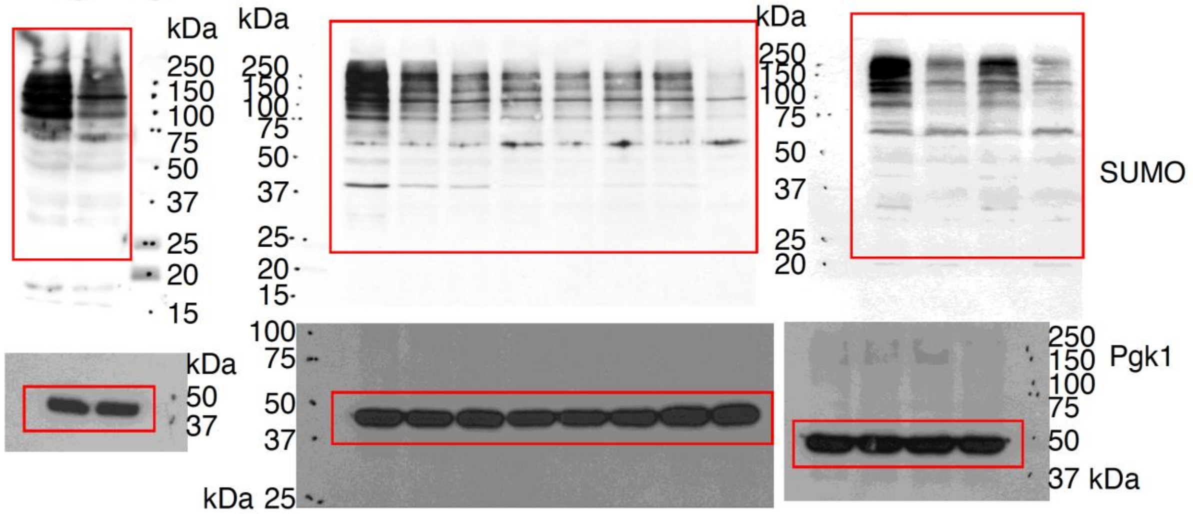


Fig. 6d

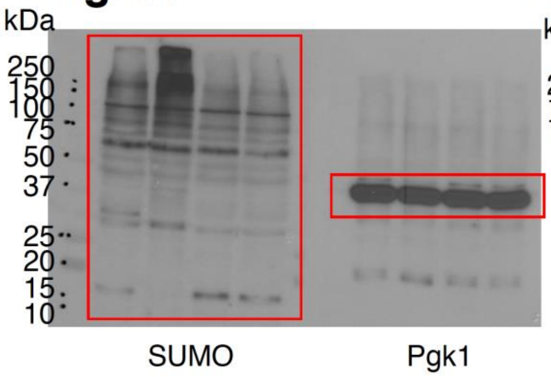


Fig. 6l

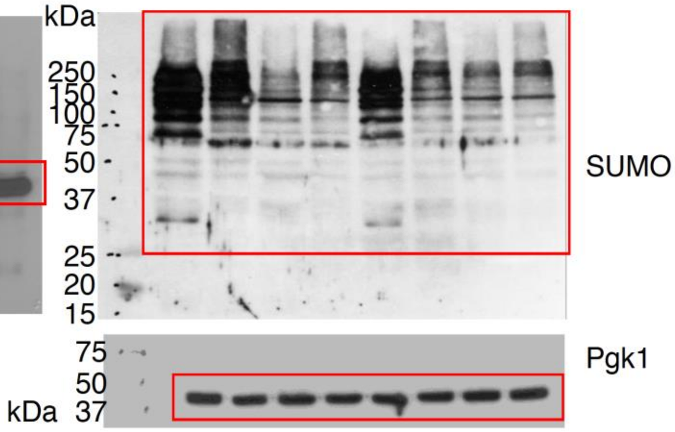


Fig. 6h

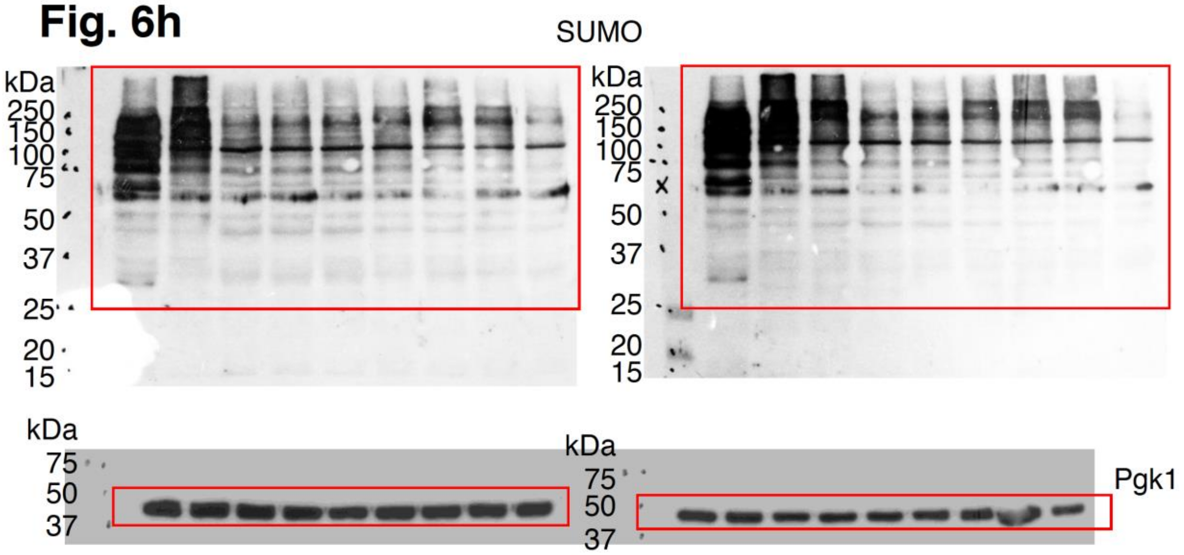

## Supplementary Fig. 1b

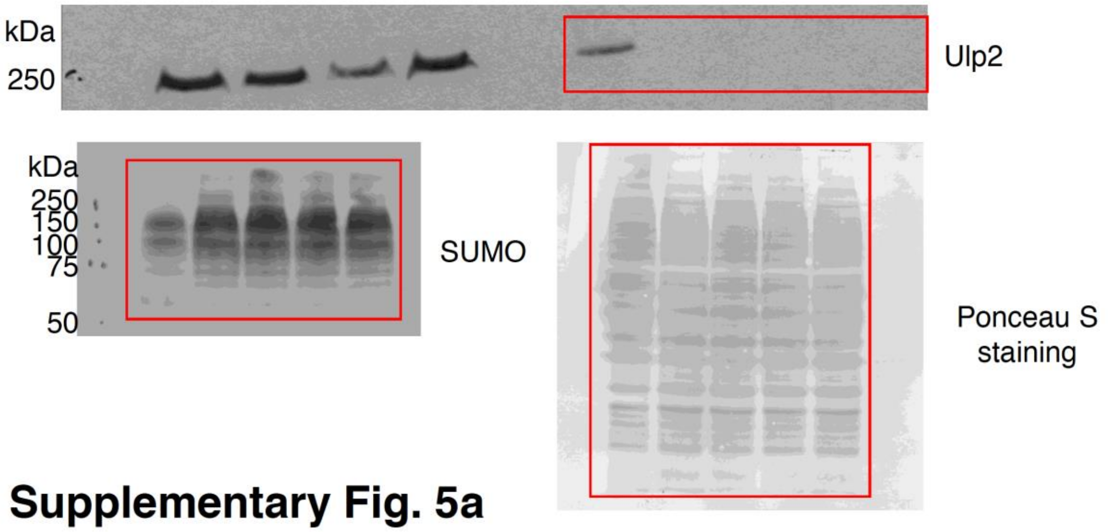


## Supplementary Fig. 5a

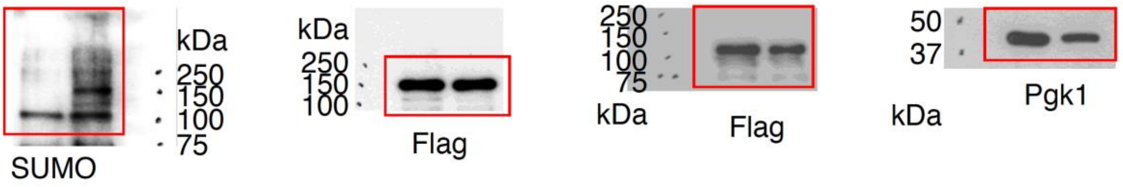


## Supplementary Fig. 6c

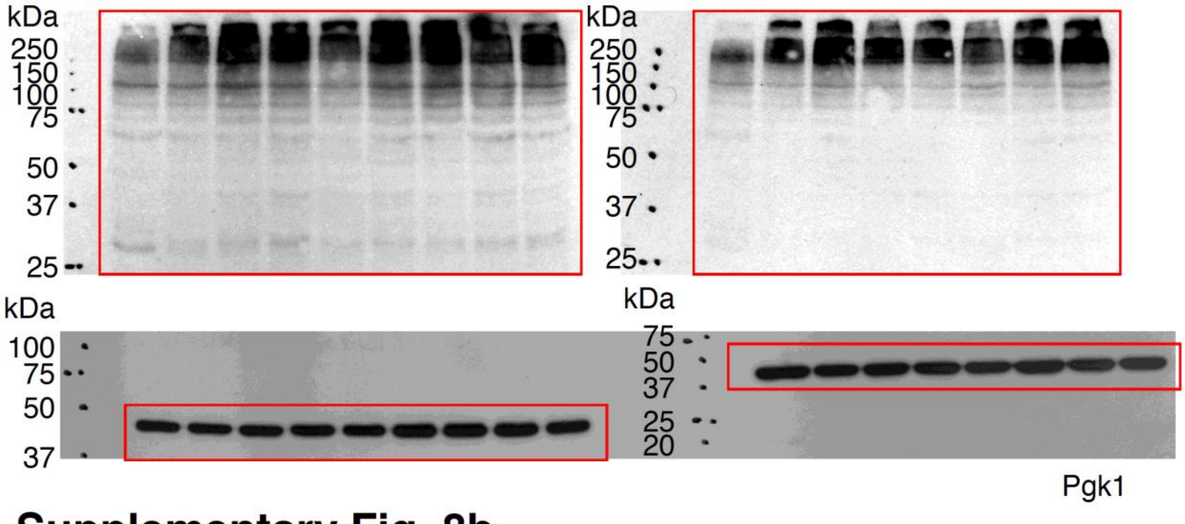


## Supplementary Fig. 8b

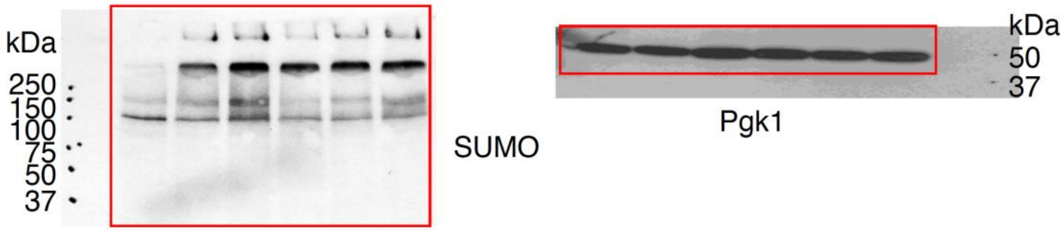

**Supplementary Tables**

**Supplementary Table 1.** Yeast strains

| Strain | Genotype | Source |
|---|---|---|
| MHY500 | *MATa his3-200 leu2-3,112 lys2-801 trp1-1 ura3-52* | [1] |
| MHY606 | *MATa*/*MATα his3-Δ200*/*his3-Δ200 leu2-3,112*/*leu2-3,112 lys2-801*/*lys2-801 trp1-1*/*trp1-1 ura3-52*/*ura3-52* | [2] |
| MHY1328 | *MATa*/*MATα his3-Δ200*/*his3-Δ200 leu2-3,112*/*leu2-3,112 lys2-801*/*lys2-801 trp1-1*/*trp1-1 ura3-52*/*ura3-52 ULP2*/*ulp2Δ::HIS3* | [3] |
| MHY1379 | *MATa his3-Δ200 leu2-3,112 lys2-801 trp1-1 ura3-52 ulp2Δ::HIS3* [YCplac33-*ULP2*] | [3] |
| MHY1538 | *MATα his3-Δ200 leu2-3,112 lys2-801 trp1-1 ura3-52 smt3Δ::HIS3 ulp1Δ::HIS3* [pVT102U-*SMT3gg*, YCp50-*ULP1*] | [4] |
| MHY1620 | *MATa his3-Δ200 leu2-3,112::LEU2::ubc9-1 ura3-52 lys2-801 trp1-1 ubc9Δ::TRP1* | [5] |
| MHY4027 (JR5-2A) | *MATa ade2-1 ura3-1 his3-11, -15 trp1-1 leu2-3,112 can1-100 ssd1 htb1-1 htb2*-1 [YCp50-*HTB1*] | [6] |
| MHY4659 | *MATα his3-Δ200 leu2-3,112 lys2-801 trp1-1 ura3-52 doa10-Δ1::HIS3 uba2Δ::KanMX6 [pIS-uba2ts]* | [5] |
| MHY5033 | *MATα his3-Δ200 leu2-3,112 lys2-801 trp1-1 ura3-52 ulp2Δ::KanMX6* | [7] |
| MHY5339 | *MATα ade2-1 ura3-1 his3-11, -15 trp1-1 leu2-3,112 can1-100 GAL-His6-SMT3::kanMX6* | [8] |
| MHY7863 | *MATa his3-Δ200 leu2-3,112 lys2-801 trp1-1 ura3-52 ULP2-6xGly-3xFlag::HIS3MX6* | [7] |
| MHY9388 | *MATa his3-Δ200 leu2-3,112 lys2-801 trp1-1 ura3-52 ccr4Δ::kanMX6* | [7] |
| MHY9389 | *MATa his3-Δ200 leu2-3,112 lys2-801 trp1-1 ura3-52 ulp2Δ::HIS3 ccr4Δ::kanMX6* | [7] |
| MHY9393 | *MATa his3-Δ200 leu2-3,112 lys2-801 trp1-1 ura3-52 ulp2Δ::HIS3 CCR4-6xGly-3xFlag::kanMX6* [YCplac33-*ULP2*] | This study |
| MHY9494 (Y911) | *MATa leu2Δ0 met15Δ0 ura3Δ0 HIS3::pRS303-ADH-AFB2 ULP2-AIDstar-6FLAG* (*+neo-HHR-B2* in 3'UTR) | This study |
| MHY9610 | *MATa his3Δ1 leu2Δ0 met15Δ0 ura3Δ0 RAP1-TAP::HIS3MX6* | TAP tag library |
| MHY9611 | *MATa his3Δ1 leu2Δ0 met15Δ0 ura3Δ0 HST3-TAP::HIS3MX6* | TAP tag library |
| MHY9612 | *MATa his3Δ1 leu2Δ0 met15Δ0 ura3Δ0 NRG2-TAP::HIS3MX6* | TAP tag library |
| MHY9613 | *MATa ade2-1 ura3-1 his3-11, -15 trp1-1 leu2-3,112 can1-100 ssd1 htb1-1 htb2-1 smt3Δ::HIS3* [pRS314-*FLAG-H2B*, pRS425-*GPD-HIS6-SMT3*] | This study |
| MHY9618 | *MATa ade2-1 ura3-1 his3-11, -15 trp1-1 leu2-3,112 can1-100 ssd1 htb1-1 htb2-1 smt3Δ::HIS3 ulp2Δ::kanMX6* | This study |

| | | |
|---|---|---|
| | [pRS314-*FLAG-H2B*, pRS425-*GPD-HIS6-SMT3*] | |
| MHY9619 | *MATα his3-Δ200 leu2-3,112 lys2-801 trp1-1 ura3-52 ulp2Δ::HIS3 ubc9Δ::TRP1* [YCplac33-*ULP2*, pRS315-*UBC9*] | This study |
| MHY9620 | *MATα his3-Δ200 leu2-3,112 lys2-801 trp1-1 ura3-52 ulp2Δ::HIS3 ubc9Δ::TRP1* [YCplac33-*ULP2*, pRS315-*ubc9K27N*] | This study |
| MHY9621 | *MATα his3-Δ200 leu2-3,112 lys2-801 trp1-1 ura3-52 ulp2Δ::HIS3 uba2Δ::kanMX6* [YCplac33-*ULP2*, pRS315-*UBA2*] | This study |
| MHY9622 | *MATα his3-Δ200 leu2-3,112 lys2-801 trp1-1 ura3-52 ulp2Δ::HIS3 uba2Δ::kanMX6* [YCplac33-*ULP2*, pRS315-*uba2C162S*] | This study |
| MHY9623 | *MATα his3-Δ200 leu2-3,112 lys2-801 trp1-1 ura3-52 ulp2Δ::HIS3 uba2Δ::kanMX6* [YCplac33-*ULP2*, pRS315-*uba2C162S H86D*] | This study |
| MHY9624 | *MATα his3-Δ200 leu2-3,112 lys2-801 trp1-1 ura3-52 ulp2Δ::HIS3 uba2Δ::kanMX6* [YCplac33-*ULP2*, pRS315-*uba2C162S,A414V*] | This study |
| MHY9676 | *MATα his3-Δ200 leu2-3,112 lys2-801 trp1-1 ura3-52 ulp2Δ::HIS3 uba2Δ::kanMX6* [YCplac33-*ULP2*, pRS315-*uba2C162S,A414P*] | This study |
| MHY9625 | *MATα his3-Δ200 leu2-3,112 lys2-801 trp1-1 ura3-52 ulp2Δ::HIS3 uba2Δ::KanMX6* [YCplac33-*ULP2*, pRS315-*uba2C162S S415C*] | This study |
| MHY9626 | *MATα his3-Δ200 leu2-3,112 lys2-801 trp1-1 ura3-52 ulp2Δ::HIS3 uba2Δ::kanMX6* [YCplac33-*ULP2*, pRS315-*uba2C162S,R421L*] | This study |
| MHY9627 | *MATα his3-Δ200 leu2-3,112 lys2-801 trp1-1 ura3-52 ulp2Δ::HIS3 uba2Δ::KanMX6* [YCplac33-*ULP2*, pRS315-*uba2C162S L494F*] | This study |
| MHY9637 | *MATα his3-Δ200 leu2-3,112 lys2-801 trp1-1 ura3-52 ubc9Δ::TRP1* [pRS315-*UBC9*] | This study |
| MHY9638 | *MATα his3-Δ200 leu2-3,112 lys2-801 trp1-1 ura3-52 ubc9Δ::TRP1* [pRS315-*ubc9K27N*] | This study |
| MHY9639 | *MATα his3-Δ200 leu2-3,112 lys2-801 trp1-1 ura3-52 uba2Δ::kanMX6* [pRS315-*UBA2*] | This study |
| MHY9640 | *MATα his3-Δ200 leu2-3,112 lys2-801 trp1-1 ura3-52 uba2Δ::kanMX6* [pRS315-*uba2C162S*] | This study |
| MHY9641 | *MATα his3-Δ200 leu2-3,112 lys2-801 trp1-1 ura3-52 uba2Δ::kanMX6* [pRS315-*uba2C162S,H86D*] | This study |
| MHY9642 | *MATα his3-Δ200 leu2-3,112 lys2-801 trp1-1 ura3-52 uba2Δ::kanMX6* [pRS315-*uba2C162S,A414V*] | This study |
| MHY9675 | *MATα his3-Δ200 leu2-3,112 lys2-801 trp1-1 ura3-52 uba2Δ::kanMX6* [pRS315-*uba2C162S,A414P*] | This study |
| MHY9643 | *MATα his3-Δ200 leu2-3,112 lys2-801 trp1-1 ura3-52 uba2Δ::KanMX6* [pRS315-*uba2C162S S415C*] | This study |
| MHY9644 | *MATα his3-Δ200 leu2-3,112 lys2-801 trp1-1 ura3-52* | This study |

| | | |
|---|---|---|
| | *uba2Δ::kanMX6* [pRS315-*uba2C162S,R421L*] | |
| MHY9645 | *MATα his3-Δ200 leu2-3,112 lys2-801 trp1-1 ura3-52 uba2Δ::kanMX6* [pRS315-*uba2C162S,L494F*] | This study |
| MHY9677 | *MATα his3-Δ200 leu2-3,112 lys2-801 trp1-1 ura3-52 ulp2Δ::HIS3 uba2Δ::kanMX6 aos1Δ::NatMX4* [YCplac33-*ULP2*, pRS315-*UBA2-AOS1*] | This study |
| MHY9678 | *MATα his3-Δ200 leu2-3,112 lys2-801 trp1-1 ura3-52 ulp2Δ::HIS3 uba2Δ::kanMX6 aos1Δ::natMX4* [YCplac33-*ULP2*, pRS315- *uba2C162S-AOS1*] | This study |
| MHY9679 | *MATα his3-Δ200 leu2-3,112 lys2-801 trp1-1 ura3-52 ulp2Δ::HIS3 uba2Δ::kanMX6 aos1Δ::natMX4* [YCplac33-*ULP2*, pRS315- *UBA2-aos1E346V*] | This study |
| MHY9680 | *MATα his3-Δ200 leu2-3,112 lys2-801 trp1-1 ura3-52 ulp2Δ::HIS3 uba2Δ::kanMX6 aos1Δ::natMX4* [YCplac33-*ULP2*, pRS315- *uba2C162S-aos1E346V*] | This study |
| MHY9681 | *MATα his3-Δ200 leu2-3,112 lys2-801 trp1-1 ura3-52 uba2Δ::kanMX6 aos1Δ::natMX4* [pRS315-*UBA2-AOS1*] | This study |
| MHY9682 | *MATα his3-Δ200 leu2-3,112 lys2-801 trp1-1 ura3-52 uba2Δ::kanMX6 aos1Δ::natMX4* [pRS315- *uba2C162S-AOS1*] | This study |
| MHY9683 | *MATα his3-Δ200 leu2-3,112 lys2-801 trp1-1 ura3-52 uba2Δ::kanMX6 aos1Δ::natMX4* [pRS315-*UBA2-aos1E346V*] | This study |
| MHY9684 | *MATα his3-Δ200 leu2-3,112 lys2-801 trp1-1 ura3-52 uba2Δ::kanMX6 aos1Δ::natMX4* [pRS315- *uba2C162S-aos1E346V*] | This study |
| MHY10229 | *MATa his3-Δ200 leu2-3,112 lys2-801 trp1-1 ura3-52 ulp2Δ::HIS3 NOT5-6xGly-3xFlag::kanMX6* [YCplac33-*ULP2*] | This study |
| MHY10230 | *MATa his3-Δ200 leu2-3,112 lys2-801 trp1-1 ura3-52 ulp2Δ::HIS3 RAP1-6xGly-3xFlag::kanMX6* [YCplac33-*ULP2*] | This study |

**Supplementary Table 2.** Plasmids

| Plasmid | Description | Source |
|---|---|---|
| YCplac33 | *CEN, URA3* | [9] |
| YCplac33-ULP2 | *CEN, URA3, ULP2* | [3] |
| YCplac33-UBC9 | *CEN, URA3, UBC9* | Alaron Lewis |
| YCplac33-UBA2 | *CEN, URA3, UBA2* | Alaron Lewis |
| YCplac33-UBA2 AOS1 | *CEN, URA3, UBA2, AOS1* | This study |
| pGP564 | *2μ, LEU2* | [10] |
| pGP564-REX3+snR6+YLR108C | *2μ, LEU2, REX3, snR6, YLR108C* | This study |
| pGP564-AHP1 | *2μ, LEU2, AHP1* | This study |
| pGP564-CCW12 | *2μ, LEU2, CCW12* | This study |
| pGP564-YLR111W | *2μ, LEU2, YLR111W* | This study |
| pGP564-YLR112W+HOG1 | *2μ, LEU2, YLR112W, HOG1* | This study |
| pGP564-tR(ACG)L+AVL9 | *2μ, LEU2, tR(ACG)L, AVL9* | This study |
| pGP564-REC102 | *2μ, LEU2, REC102* | This study |
| pGP564-CHS5 | *2μ, LEU2, CHS5* | This study |
| pGP564-JIP3+MID2 | *2μ, LEU2, JIP3, MID2* | This study |
| pGP564-tD(GUC)L2+snoRNAs | *2μ, LEU2, tD(GUC)L2, snR61, snR55, snR57* | This study |
| pGP564-snoRNAs | *2μ, LEU2, snR61, snR55, snR57* | This study |
| pGP564-RPS25B | *2μ, LEU2, RPS25B* | This study |
| pGP564-YLR334C+tE(UUC)L+NUP2 | *2μ, LEU2, YLR334C, tE(UUC)L, NUP2* | This study |
| pGP564-SGD1 | *2μ, LEU2, SGD1* | This study |
| pGP564-VRP1+OPI9 | *2μ, LEU2, VRP1, OPI9* | This study |
| pGP564-YLR339C+RPP0+SPO77 | *2μ, LEU2, YLR339C, RPP0, SPO77* | This study |
| pRS315 | *CEN, LEU2* | [11] |
| pRS315-UBC9 | *CEN, LEU2, UBC9* | This study |
| pRS315-UBC9K27N | *CEN, LEU2, ubc9K27N* | This study |
| pRS315-UBA2 | *CEN, LEU2, UBA2* | This study |
| pRS315-UBA2C162S | *CEN, LEU2, uba2C162S* | This study |
| pRS315-UBA2C162S H86D | *CEN, LEU2, uba2C162S,H86D* | This study |
| pRS315-UBA2C162S A414V | *CEN, LEU2, uba2C162S,A414V* | This study |
| pRS315-UBA2C162S A414P | *CEN, LEU2, uba2C162S,A414P* | This study |
| pRS315-UBA2C162S S415C | *CEN, LEU2, uba2C162S,S415C* | This study |
| pRS315-UBA2C162S R421L | *CEN, LEU2, uba2C162S,R421L* | This study |
| pRS315-UBA2C162S L494F | *CEN, LEU2, uba2C162S,L494F* | This study |
| pRS315-UBA2-AOS1 | *CEN, LEU2, UBA2, AOS1* | This study |
| pRS315-UBA2C162S-AOS1 | *CEN, LEU2, uba2C162S, AOS1* | This study |
| pRS315-UBA2-AOS1E346V | *CEN, LEU2, UBA2, aos1E346V* | This study |
| pRS315-UBA2C162S-AOS1E346V | *CEN, LEU2, uba2C162S, aos1E346V* | This study |
| p425-GPD | *2μ, LEU2*, $P_{GPD}$, $T_{CYC1}$ | [12] |
| p425-GPD-CLN3 | *2μ, LEU2*, $P_{GPD}$, $T_{CYC1}$, *CLN3* | [7] |
| p425-GPD-CCR4 | *2μ, LEU2*, $P_{GPD}$, $T_{CYC1}$, *CCR4* | [7] |
| p425-GPD-NOT5 | *2μ, LEU2*, $P_{GPD}$, $T_{CYC1}$, *NOT5* | [7] |

| | | |
|---|---|---|
| p425-GPD-HIS6-SMT3 | *2μ*, *LEU2*, $P_{GPD}$, $T_{CYC1}$, *HIS6-SMT3* | This study |
| pRS314-FLAG-H2B | *CEN*, *TRP1*, *FLAG-HTB1* | [13] |

**Supplementary Table 3.** Oligonucleotides

| Name | Sequence |
|---|---|
| ChrI_Left_FWD | ACAGCTTCTAAACGTTCCGTGTGC |
| ChrI_Left_REV | GCGGTGTGTGGATGATGGTTTCAT |
| ChrI_Right_FWD | GCACTTGATCCATGTAGCCATACTCG |
| ChrI_Right_REV | TTCGGGTGACCCTTATGGCATTCT |
| ChrV_Left_FWD | TCCGCCGGCAACTGTAACTGTAAA |
| ChrV_Left_REV | ATAGTAACCAACGAGAGCGCGCAA |
| ChrV_Right_FWD | CAAGCCACTGTTGGCGTTTCAACT |
| ChrV_Right_REV | TTTATGTGCGGCTTTGTCAGCAGG |
| ChrVIII_Left_FWD | TTGTCGGTCTAGCCGAAAGGTGTT |
| ChrVIII_Left_REV | AGTTCTGCGGCAGTAATGTAGGGT |
| ChrVIII_Right_FWD | TGGAAAGGGCCTCGAAAGACGTTA |
| ChrVIII_Right_REV | TCGGGACTCCACCTGGAATATTGT |
| ChrX_Left_FWD | ATTTACCGGTTAGTGTCAGCGCCA |
| ChrX_Left_REV | CGACAGAGTAGTTTATGCCGAGGGTT |
| ChrX_Right_FWD | AGGCGAGTACCCTTAGCATTTCCT |
| ChrX_Right_REV | ACGAGGCAAGTGTAGGTCCTTTGT |
| ChrXII_Left_FWD | TGGAGATGAAGGGTTGTCGTTGGT |
| ChrXII_Left_REV | ACGTGTAGCGTTTCTGCTGGTCTT |
| ChrXII_Right_FWD | ATGGCAGGCAGGTGAATGAGATGA |
| ChrXII_Right_REV | AGAGTAGACCATGGGACGTCGTTT |
| RPL12B pro For (777-800) | GCTCTAGGCCGAGAAAATGTTAAT |
| RPL12B pro Rev (884-910) | TCATACTAGTAATGTACCGCCAAAGAA |
| RPL12B CDS For (1178-1199) | ACAGAGATCCGTTTGCGTGACT |
| RPL12B CDS Rev (1248-1267) | AAAGCGGCTAGAGGGGACTG |
| RPS18B pro For (793-812) | GTCTCCGCAGGCCTCTTGTT |
| RPS18B pro Rev (869-893) | CCAGTTAGGATAGCAAAGGTGTCAG |
| RPS18B CDS For (1508-1529) | AGGGTGTTGGTCGTCGTTACTC |
| RPS18B CDS Rev (1609-1629) | TGGGTTTTGCATGATTTGGAC |
| RPS20 pro For (767-792) | AAACATAAGAGAAAAAATGCCACAAC |
| RPS20 pro Rev (887-918) | CTTTACTATTATTTTGAATTCAATACGGTCTA |
| RPS20 CDS For (1202-1225) | AGAAAGACTCCAAATGGTGAAGGT |
| RPS20 CDS Rev (1242-1265) | TCTTGTGGATTCTCATTTCGTAGG |
| snR30 pro For (807-828) | AGTAACCCTAAACCATCAGCGG |
| snR30 pro Rev (924-947) | CCATAGAAGTTAAATGCACGACGA |
| snR30 CDS For (1361-1379) | CAGAAGTGGCCCCGTTGAC |
| snR30 CDS Rev (1457-1480) | ACATCCCGCAATCTCTTCGTAATA |
| snR40 pro For (773-779) | TTCTTCTTAAAGTATAAAAGGCGGACA |
| snR40 pro Rev (875-896) | ACCGGTTCGCACTACGATACTT |
| snR40 CDS For (1006-1027) | TGACGAGAAAAAAGCTGTGCAC |
| snR40 CDS Rev (1068-1095) | TCAGAAATTTGGGTATACTTAATGCTTC |
| snR60 pro For (759-779) | ACTTCGTCGCTTTCTCCTCCA |
| snR60 pro Rev (877-899) | AGGCATACGGCAAATTGACTACA |
| snR60 CDS For (1002-1030) | GTTAATGATGATAACCAAAGATGCATAGT |
| snR60 CDS Rev (1077-1103) | TTCAGATAGGAGCGAAAGACTAATTTC |

| | |
|---|---|
| SNR57 Pro For (650-675) | ATTGAACCCTAACAAATGCTACAGTG |
| SNR57 Pro Rev (752-781) | CATCCAACTCAATTAAGAAAGTTAGAAGAT |
| SNR57 ORF For (1076-1100) | TTCGTTTATGATCTGGCCTCTTTAT |
| SNR57 ORF Rev (1139-1164) | TTGAACCGGATATTTTAATCAGTGTC |
| 18S For | GCTTGCGTTGATTACGTCCC |
| 18S Rev | CACTAAGCCATTCAATCGGT |
| 25S For | CGTTCATAGCGACATTGCTT |
| 25S Rev | GGGTGAACAATCCAACGCTT |
| SPT15 For | TAAAAAGAGCTGCCCCAGAA |
| SPT15 Rev | ATGATGACAGCAGCAAAACG |
| ACT1_qRT-PCR_FWD | GACGCTCCTCGTGCTGTCTTC |
| ACT1_qRT-PCR_REV | GAGCTTCATCACCAACGTAGGAGTC |
| Int IV For (1516109-1516129) | CGCATTACCAGACGGAGATGT |
| Int IV Rev (1516212-1516234) | CAAGCAAGCCTTGTGCATAAGA |

**Supplementary References**


1. Chen P, Johnson P, Sommer T, Jentsch S, Hochstrasser M. Multiple Ubiquitin-Conjugating Enzymes Participate in the in-Vivo Degradation of the Yeast Mat-Alpha-2 Repressor. *Cell* **74**, 357-369 (1993).

2. Papa FR, Hochstrasser M. The yeast DOA4 gene encodes a deubiquitinating enzyme related to a product of the human tre-2 oncogene. *Nature* **366**, 313-319 (1993).

3. Li SJ, Hochstrasser M. The yeast ULP2 (SMT4) gene encodes a novel protease specific for the ubiquitin-like Smt3 protein. *Mol Cell Biol* **20**, 2367-2377 (2000).

4. Hannich JT*, et al.* Defining the SUMO-modified proteome by multiple approaches in Saccharomyces cerevisiae. *J Biol Chem* **280**, 4102-4110 (2005).

5. Xie Y, Rubenstein EM, Matt T, Hochstrasser M. SUMO-independent in vivo activity of a SUMO-targeted ubiquitin ligase toward a short-lived transcription factor. *Gene Dev* **24**, 893-903 (2010).

6. Robzyk K, Recht L, Osley MA. Rad6-dependent ubiquitination of histone H2B in yeast. *Science* **287**, 501-504 (2000).

7. Ryu HY, Wilson NR, Mehta S, Hwang SS, Hochstrasser M. Loss of the SUMO protease Ulp2 triggers a specific multichromosome aneuploidy. *Genes Dev* **30**, 1881-1894 (2016).

8. Felberbaum R, Wilson NR, Cheng DM, Peng JM, Hochstrasser M. Desumoylation of the Endoplasmic Reticulum Membrane VAP Family Protein Scs2 by Ulp1 and SUMO Regulation of the Inositol Synthesis Pathway. *Molecular and Cellular Biology* **32**, 64-75 (2012).

9. Gietz RD, Sugino A. New yeast-Escherichia coli shuttle vectors constructed with in vitro mutagenized yeast genes lacking six-base pair restriction sites. *Gene* **74**, 527-534 (1988).

10. Jones GM*, et al.* A systematic library for comprehensive overexpression screens in Saccharomyces cerevisiae. *Nat Methods* **5**, 239-241 (2008).

11. Sikorski RS, Hieter P. A system of shuttle vectors and yeast host strains designed for efficient manipulation of DNA in Saccharomyces cerevisiae. *Genetics* **122**, 19-27 (1989).

12. Mumberg D, Muller R, Funk M. Yeast vectors for the controlled expression of heterologous proteins in different genetic backgrounds. *Gene* **156**, 119-122 (1995).

13. Nathan D*, et al.* Histone sumoylation is a negative regulator in Saccharomyces cerevisiae and shows dynamic interplay with positive-acting histone modifications. *Genes Dev* **20**, 966-976 (2006).
