# Supplementary material for: Distinct adaptive mechanisms drive recovery from aneuploidy caused by loss of the Ulp2 SUMO protease
Source: Nat Commun. 2018 Dec 21;9:5417. doi: 10.1038/s41467-018-07836-0 (PMC6303320; doi:10.1038/s41467-018-07836-0)
Supplement: Supplementary file 3 — Description of Additional Supplementary Files [file 41467_2018_7836_MOESM3_ESM.pdf]

Description of Additional Supplementary Files

File Name: Supplementary Data 1
Description: Gene Ontology analysis of ulp2Δ cells during passaging
This is the list of genes categorized by GO biological process in Figure 2C.

File Name: Supplementary Data 2
Description: Expression of snoRNA genes in high-passage ulp2Δ cells
Transcript levels of snoRNAs in high-passage ulp2Δ cells were compared to the levels in MHY1379 (ulp2Δ + YCplac33-ULP2) at 50G.
