# Supplementary material for: Distinct adaptive mechanisms drive recovery from aneuploidy caused by loss of the Ulp2 SUMO protease
Source: Nat Commun. 2018 Dec 21;9:5417. doi: 10.1038/s41467-018-07836-0 (PMC6303320; doi:10.1038/s41467-018-07836-0)
Supplement: Supplementary file 6 — Reporting Summary [file 41467_2018_7836_MOESM6_ESM.pdf]

nature research

Corresponding author(s): Mark Hochstrasser

Reporting Summary

Nature Research wishes to improve the reproducibility of the work that we publish. This form provides structure for consistency and transparency in reporting. For further information on Nature Research policies, see Authors & Referees and the Editorial Policy Checklist.

Statistical parameters

When statistical analyses are reported, confirm that the following items are present in the relevant location (e.g. figure legend, table legend, main text, or Methods section).

n/a	Confirmed	
☐	☒	The exact sample size (*n*) for each experimental group/condition, given as a discrete number and unit of measurement
☐	☒	An indication of whether measurements were taken from distinct samples or whether the same sample was measured repeatedly
☐	☒	The statistical test(s) used AND whether they are one- or two-sided<br>*Only common tests should be described solely by name; describe more complex techniques in the Methods section.*
☒	☐	A description of all covariates tested
☐	☒	A description of any assumptions or corrections, such as tests of normality and adjustment for multiple comparisons
☐	☒	A full description of the statistics including central tendency (e.g. means) or other basic estimates (e.g. regression coefficient) AND variation (e.g. standard deviation) or associated estimates of uncertainty (e.g. confidence intervals)
☐	☒	For null hypothesis testing, the test statistic (e.g. *F*, *t*, *r*) with confidence intervals, effect sizes, degrees of freedom and *P* value noted<br>*Give P values as exact values whenever suitable.*
☒	☐	For Bayesian analysis, information on the choice of priors and Markov chain Monte Carlo settings
☐	☒	For hierarchical and complex designs, identification of the appropriate level for tests and full reporting of outcomes
☒	☐	Estimates of effect sizes (e.g. Cohen's *d*, Pearson's *r*), indicating how they were calculated
☐	☒	Clearly defined error bars<br>*State explicitly what error bars represent (e.g. SD, SE, CI)*

Our web collection on statistics for biologists may be useful.

Software and code

Policy information about availability of computer code

Data collection	Tophat v2.1.1 and Cufflinks v2.2.1 were used for analysis of RNA-seq assay. BWA MEM v0.7.15 and freebayes v0.9.14 were used for DNA-seq analysis
Data analysis	Gene Ontology (GO) enrichment analysis of the genes displayed in Figure 2b. Bar diagrams indicate the fold-enrichment of categories of biological process to the genome using GO data from SGD and PANTHER. The genes used in GO biological process are listed in Supplementary File 1.

For manuscripts utilizing custom algorithms or software that are central to the research but not yet described in published literature, software must be made available to editors/reviewers upon request. We strongly encourage code deposition in a community repository (e.g. GitHub). See the Nature Research guidelines for submitting code & software for further information.

April 2018

Data

Policy information about availability of data

All manuscripts must include a data availability statement. This statement should provide the following information, where applicable:

- Accession codes, unique identifiers, or web links for publicly available datasets
- A list of figures that have associated raw data
- A description of any restrictions on data availability

The RNA-seq and DNA-seq data used in this publication have been deposited in Gene Expression Omnibus (GEO) with accession GSE121898 (https://www.ncbi.nlm.nih.gov/geo/query/acc.cgi?acc=GSE121898) and GSE121899 (https://www.ncbi.nlm.nih.gov/geo/query/acc.cgi?acc=GSE121899), respectively.

Field-specific reporting

Please select the best fit for your research. If you are not sure, read the appropriate sections before making your selection.

☒ Life sciences ☐ Behavioural & social sciences ☐ Ecological, evolutionary & environmental sciences

For a reference copy of the document with all sections, see nature.com/authors/policies/ReportingSummary-flat.pdf

Life sciences study design

All studies must disclose on these points even when the disclosure is negative.

Sample size	Sample size was considered to get the significant results by two-tailed Student's t test (P < 0.05, P < 0.01).
Data exclusions	In RNA-seq resuls, only significantly changed genes were analyzed for further experiments and those are described detailedly in the manuscript.
Replication	All of results were verified by repeated experiments.
Randomization	We do not need randomization, because we used yeast model organism system.
Blinding	We do not need blinding. Instead of it, we did enough repeated experiments to get the significant results.

Reporting for specific materials, systems and methods

Materials & experimental systems

n/a	Involved in the study
☐	☒ Unique biological materials
☐	☒ Antibodies
☐	☒ Eukaryotic cell lines
☒	☐ Palaeontology
☒	☐ Animals and other organisms
☒	☐ Human research participants

Methods

n/a	Involved in the study
☒	☐ ChIP-seq
☐	☒ Flow cytometry
☒	☐ MRI-based neuroimaging

Unique biological materials

Policy information about availability of materials

Obtaining unique materials	Ni-NTA agarose beads (R90115, Thermo Scientific), iQ SYBR Green Supermix kit (Biorad), indole-3-acetic acid (SIGMA), neomycin (SIGMA), rapamycin (Enzo Life Sciences), RNeasy kit (Qiagen), iScript cDNA synthesis kit (Bio-Rad), and DNA-free DNA removal kit (Ambion)

Antibodies

Antibodies used	IgG-Sepharose beads (17-0969-01, GE Healthcare), anti-Flag agarose beads (A2220, Sigma), anti-Flag (F3165, SIGMA), anti-Pgk1 (459250, Molecular Probes), anti-SUMO (Hochstrasser lab) and anti-Rpl32 antibody (PA5-69070, Invitrogen)

Validation	IgG-Sepharose beads: IgG Sepharose 6 Fast Flow uses the rigid Sepharose 6 Fast Flow matrix, covalently coupled with human IgG, to purify protein A-containing proteins.<br>anti-Flag agarose beads: ANTI-FLAG® M2 Affinity Agarose Gel, Anti-ddddk, Anti-dykddddk, Monoclonal ANTI-FLAG® M2 antibody produced in mouse.<br>anti-Flag: This monoclonal antibody is produced in mouse and recognizes the FLAG sequence at the N-terminus, Met N-terminus, and C-terminus.<br>anti-Pgk1: Immunogen - Yeast pGK (3-Phosphoglyceric Phosphokinase), EC 2.7.2.3 & Host/Isotype - Mouse / IgG1, kappa<br>anti-SUMO (rabbit polyclonal IgG) was purified in Hochstrasser lab and it was used in (SJ Li, M Hochstrasser - Nature, 1999).<br>anti-Rpl32 antibody: Immunogen - synthetic peptide directed towards the middle region of RPL32 & Host/Isotype - Rabbit / IgG

Eukaryotic cell lines

Policy information about cell lines

Cell line source(s)	MHY500 and BY4741; both backgrounds of yeast strains are generally used in this field.
Authentication	MHY500 background cells have verified repeatedly in Hochstrasser lab and BY4741 background cells were purchased in Open biosystems.
Mycoplasma contamination	we could not find any contamination.
Commonly misidentified lines<br>(See ICLAC register)	absence

Flow Cytometry

Plots

Confirm that:

☒ The axis labels state the marker and fluorochrome used (e.g. CD4-FITC).

☒ The axis scales are clearly visible. Include numbers along axes only for bottom left plot of group (a 'group' is an analysis of identical markers).

☒ All plots are contour plots with outliers or pseudocolor plots.

☐ A numerical value for number of cells or percentage (with statistics) is provided.

Methodology

Sample preparation	Yeast cells in exponential growth were fixed in 70% ethanol, digested with RNase A, and then stained with propidium iodide at 50 µg/ml final concentration.
Instrument	FACS LSRII flow cytometer (BD Biosciences) with a green laser (150 mW at 532 nm)
Software	FlowJo ver.8
Cell population abundance	Cell population was divided by PI signals indicating the number of chromosomes.
Gating strategy	Gating boundaries were designed equally in all of analyses.

☐ Tick this box to confirm that a figure exemplifying the gating strategy is provided in the Supplementary Information.
